# Clozapine Augments Delta, Theta, and Right Frontal EEG Alpha Power in Schizophrenic Patients

**DOI:** 10.5402/2012/596486

**Published:** 2012-05-10

**Authors:** D. MacCrimmon, D. Brunet, M. Criollo, H. Galin, J. S. Lawson

**Affiliations:** ^1^Department of Psychiatry, McMaster University, Hamilton, ON, Canada L8S 4L8; ^2^St. Joseph's Mountain Healthcare Services, 100 West 5th Street, Hamilton, ON, Canada L8N 3K7; ^3^Department of Medicine, Queen's University, Kingston, ON, Canada K7L 4Z4; ^4^QEEG Assessment Services Inc., Thornhill, ON, Canada L3T 4Z4; ^5^Department of Psychiatry, Queen's University, Kingston, ON, Canada K7L 4Z4

## Abstract

*Objective*. To explore the Quantitative EEG (QEEG) effects of established clozapine therapy regimes compared to those of previous ineffective antipsychotic regimes among 64 chronic (DSM-IV) schizophrenic patients. *Methods*. Data from 20 EEG channels referenced to linked ears were collected before and during maintenance clozapine therapy (mean duration 1.4 years). Absolute power was calculated in six frequency bands: delta (0.4–3.6 Hz), theta (4.2–7.8 Hz), alpha (8.2–11.8 Hz), beta1 (12.2–15.8 Hz), beta2 (16.2–19.8 Hz), and beta3 (20.2–23.8 Hz). *Results*. Clozapine augments power globally in the delta and theta bands, but this effect is more pronounced over frontal areas. Beta3 power was reduced. Alpha showed a frontal increase, more pronounced in the right, coupled with a posterior decrease with no net change in overall power. *Conclusion*. The demonstration of a significant clozapine-induced alpha topographic shift frontally and to the right is a novel discovery that may serve to encourage further investigations of subcortical structures in attempts to better understand the diverse aetiologies and optimal treatments of the schizophrenias.

## 1. Introduction

Schizophrenic patients have been the subjects of electroencephalographic studies since the pioneering work of Berger in 1929 [1]. The consensus in conventional EEG literature is that nonspecific abnormalities are found to be more frequent among schizophrenic patients with incidences ranging from 10 to 80 percent. Quantitative EEG studies of schizophrenic patients compared to normals generally report differences between these groups, but the nature of these differences is inconsistent from one study to the next. Statistical issues such as small sample sizes and technical differences such as number of leads, references chosen, differences in band definition, and the nature of reported data (e.g., relative power, absolute power or ratios) make comparisons among studies difficult.

As in the case of QEEG studies of drug-free schizophrenic patients, the field of medication-induced changes is similarly lacking in consistency. In one of the larger studies reported to date, John et al. [2] report QEEG data derived from 130 schizophrenic patients compared to 162 normals. First break, never medicated schizophrenics (*N* = 15) were characterized as showing no delta abnormality, a right hemisphere excess theta most pronounced over the right frontal area, significant alpha excess most pronounced over frontal and central regions, and a beta excess, which like theta was maximal over the frontal areas. Drug-free (*N* = 25) and medicated (*N* = 94) chronic patients showed similar patterns of absolute power: posterior deficit of delta activity, a frontal theta excess, a posterior alpha deficiency, and a more pronounced posterior deficit in beta. Frontal excess delta was seen only in the nonmedicated group. From these data, contrasting never medicated, chronic currently unmedicated, and chronic medicated patients, one could infer that chronic conventional neuroleptic treatment might be expected to reduce frontal delta in established unmedicated cases but would have little effect on excessive frontal theta or reduced posterior alpha. A further reduction in posterior beta power might also be seen. These results accord with a number of smaller studies [3, 4].

Clozapine is arguably unique among the so-called atypical antipsychotic agents because of superior therapeutic efficacy [5] and has been the focus of a small number of QEEG studies. Small et al. [6] studied chronic treatment resistant patients (27 men, 7 women) in the context of a double-blind comparison of placebo, haloperidol, chlorpromazine (CPZ), and clozapine. Their data demonstrated increased frontal delta combined with significant increases in theta over anterior regions, particularly with clozapine and chlorpromazine. Extending their work, these authors [7] studied 29 chronic, treatment resistant schizophrenic patients after six weeks of therapy. They replicated their earlier observations that delta activity was higher in frontal regions particularly with both chlorpromazine and clozapine but showed in addition that clozapine caused significantly increased frontal theta. No other differences were found, and, in particular, there were no changes in alpha amplitudes. These observations can be contrasted with the data of Galderisi et al. [8] who report the first multilead QEEG studies of healthy adults aimed at characterizing the QEEG profile of clozapine. Their subjects were 16 healthy males with a mean age of 26 years who in a double-blind placebo control design in which subjects received a single oral dose of either placebo or clozapine of 0.36 mg·per kilogram. In comparison to placebo, their six-hour clozapine QEEG data indicated a widespread increase of delta and theta and slower alpha (7.7–9.5 Hz) particularly over posterior regions, together with a generalized decrease in faster alpha (9.7–12.5 Hz). Beta activity tended to be lowered, significantly in the beta2 range (15.2–26 Hz) over most leads. Lacroix et al. [9] studied 20 treatment refractory schizophrenic patients comparing QEEG data while on conventional antipsychotics and after approximately six weeks on clozapine. They did not report delta but described increased amplitude on all 19 scalp electrodes, for theta (4–7 Hz) and alpha (8–12 Hz), whereas beta1 (13–18 Hz) showed only a centrally localized increase.

Jin et al. [10] compared 23 chronic schizophrenic patients on 5-week courses of haloperidol and clozapine with 18 on placebo, using a photic driving paradigm. They found reliable theta increase for clozapine versus placebo and decreased beta for both drugs over placebo. They also reported an increase in photic driving for theta and low alpha only with clozapine and that this effect involved primarily frontal and mid-centroparietal areas.

Joutsiniemi et al. [11] studied 42 chronic schizophrenic inpatients, 21 of whom were on clozapine while the other 21 were receiving chronic conventional therapy along with 29 healthy volunteer control subjects. The clozapine group showed a significant increase in delta and theta power over all electrodes as compared to both the conventional therapy group and the controls. These increases were characterized as most prominent at frontal, central, and parietal locations. The nonclozapine patients and their healthy controls did not differ significantly. Knott et al. [12] report on one female and 12 male patients following six weeks of clozapine treatment compared to data obtained after a three-day washout from conventional therapy. This important within-subjects study showed that, at six weeks, clozapine significantly increased absolute power at all sites for both delta (1.5–3.5 Hz) and theta (3.5–7.5 Hz), while the beta band (12.5–25 Hz) showed significant localized increase centred on the vertex. There were no changes in absolute alpha (7.5–12.5 Hz).

These earlier studies of the effects of CLZ were based on small sample sizes ranging from 13 to 34 cases. The number of dependent variables per frequency band in a QEEG study is equal to the number of cranial electrode sites used within each frequency band. Any hypothesis about the overall shape of the topographic map (the response surface) requires arithmetically at least as many subjects as electrode sites, and, from practical considerations of statistical power (having a reasonable chance of rejecting the null hypothesis when it is false), several times as many subjects as electrodes. Furthermore, Lawson et al. [13] observe that the multivariate statistical models used to test for differences in the morphology of topographic maps and their various coronal and sagittal sections are not robust when applied to QEEG multivariate data. These authors do, however, propose nonparametric computationally intensive methods designed to obviate this problem.

We had the opportunity to apply these analytic methods to QEEG data obtained in the context of a preexisting clinical programme monitoring the effects of CLZ in refractory chronic schizophrenic patients.

## 2. Methods

### 2.1. Subjects

Sixty-four subjects (M/F: 39/25), comprising both in-patients and out-patients, were recruited from the Schizophrenia Service of the Hamilton Psychiatric Hospital, Hamilton, Ontario. All subjects met both DSM-IV criteria for schizophrenia and the Kane [14] criteria for treatment resistance. The study was approved by the relevant Research Ethics Committee, and all subjects gave informed consent. QEEG data were collected prior to starting clozapine treatment when patients were receiving conventional agents. A second QEEG study was done when the treating physician decided that maintenance Clozapine treatment was warranted. Because of clozapine's potentially dangerous side-effects, subjects deemed to be nonresponders were promptly discontinued and therefore were not available for inclusion in this study.

### 2.2. Treatment

When first seen, subjects were either inpatients or outpatients and were receiving a wide range of psychopharmacological agents. Although all were on at least one antipsychotic, 22 patients were on two and 3 patients were on three. Adjunctive antidepressants were used in 15, benzodiazepines in 27, and antiepileptic/mood stabilizers in 14. Usual local clinical practice entails a gradual increase in clozapine doses together with stepwise reductions of other agents as clinical assessments dictate. At the time of the second QEEG study, in addition to clozapine, 10 were receiving other antipsychotics, 22 were receiving antidepressants, 25, benzodiazepines, and 15 antiepileptic/mood stabilizers.

### 2.3. QEEG Data

A total of ten and a half minutes of eyes-closed alert data were collected in three separate three and a half minute runs using the QSI9500 system. Twenty EEG channels placed in the 10/20 configuration referenced to linked ears with impedances below 5 kOhm were collected with band pass filters at 0.5−100 Hz with a 60 Hz notch filter. Muscle, cardiac, and orbital-canthus eye movements were recorded on four separate channels. Data were digitized at an effective rate of 204.8 Hz. Stored data were independently reviewed by two experienced artifactors who eliminated segments severely contaminated by eye movement, muscle, or electrode artifacts. An overall average of 367.3 seconds (range 25–605 seconds) was accepted per subject. An FFT algorithm derived spectral estimates at 0.4 Hz increments from 2.5 second epochs of accepted data which was used to compute the absolute power in six frequency bands: Delta (0.4–3.6 Hz), theta (4.2–7.8 Hz), alpha (8.2–11.8 Hz), beta1 (12.2–15.8 Hz), beta2 (16.2–19.8 Hz), and beta3 (20.2–23.8 Hz).

Thus, the 20 power values could be transformed into a bivariate polynomial in *x* (the transverse axis) and *y* (the longitudinal axis) which exactly reproduces the original 20 observations. It should be noted that this is not, strictly speaking, a “model” of the data, whereby one seeks to provide an approximate representation of the data (excluding noise) using fewer parameters than original observations. There are 20 parameters in the model, and these parameters contain all of the information in the original 20 observations and nothing more or less. The advantage of such a transformation of the original data is that substitutions for *y* in the equation produce polynomials in *x* describing various coronal sections, and integration of the equation over *y* produces a polynomial describing average or pooled coronal sections. Similar transformations, substituting for *x*, produce analogous representations of sagittal sections.

The polynomial representation of a response surface such as an EEG topographic map has particular advantages for inferential statistical testing of differences between response surfaces under different conditions. It is not, however, a particularly good interpolant [15], and it is therefore important to confine calculations within this perimeter of observed values beyond which the polynomial tends to misbehave. Accordingly, integration of the response surface to derive an expression depicting an “average” coronal or sagittal section was confined to a circle with center at Cz (the Cartesian origin) and radius 2 units which passes through cranial sites Fp1, Fp2, F7, F8, T3, T4, P3, P4, O1, O2, Oz.

Hotelling's *T* ^2^ statistic was used to test the hypothesis that the vector of polynomial coefficients describing the shape of a particular map or section was identical in the pre- and post-CLZ conditions. The *T* ^2^ statistics follows a multiple of the F distribution when the null hypothesis is true, if the variables are multivariate normal (MVN). Lawson et al. [16] have shown that QEEG data are far from MVN and that this can cause parametric *F*-tests to be grossly misleading. Thus, we used the method of bootstrap resampling [17], a computationally intensive nonparametric method which makes no assumptions about the underlying distributions and generates the null hypothesis distribution from the observed data.

## 3. Results

1. The clinical descriptive characteristics of the patient group are given in Table 1.
2. Post-pre QEEG changes.

The *P* values in Figures 2 and 3 refer to a nonparametric bootstrap resampling [17] statistical test of the null hypothesis of congruence (same shape) of the pre- and posttopographic maps: that is, that any changes in the log power values as a result of treatment are evenly distributed over the cranial surface: that is to say that the difference map is, statistically speaking, a plane [16] (see the appendix). The contour lines connect points of equal log power on the map. The density of contour lines is proportional to the rate of change of log power differences over local areas of the cranium. The *P* values indicate the probability that the observed sample map of differences could be generated by a population in which the map differences were a plane (i.e., the null hypothesis). If *P* is small (i.e., <0.05), then the null hypothesis of unchanged map shape after clozapine becomes untenable. It can be seen from examination of Figure 3 and the associated *P* values that these power changes are not evenly distributed over the cranial surface for delta through beta1 but, as indicated by the contour line density, appear to be greater in the anterior areas. In particular, the alpha map displays both a right anterior increase in log power.

Figure 3 shows the postminus prelog power differences as topographic maps.

Red shading indicates an increase in power after treatment (positive difference), while blue shading means decreased power (negative difference). The intensity of colour is proportional to the size of the power difference. There is a global log power increase after treatment in the lowest two frequency bands, particularly in the theta band (predominantly red), while there is a decline in log power in the higher frequency bands, particularly in beta3 (not shown).

Figure 4 shows coronal and sagittal sections for only the alpha band difference map. Both pooled (i.e., averaged) and specific sections are shown.

**Figure 1:**
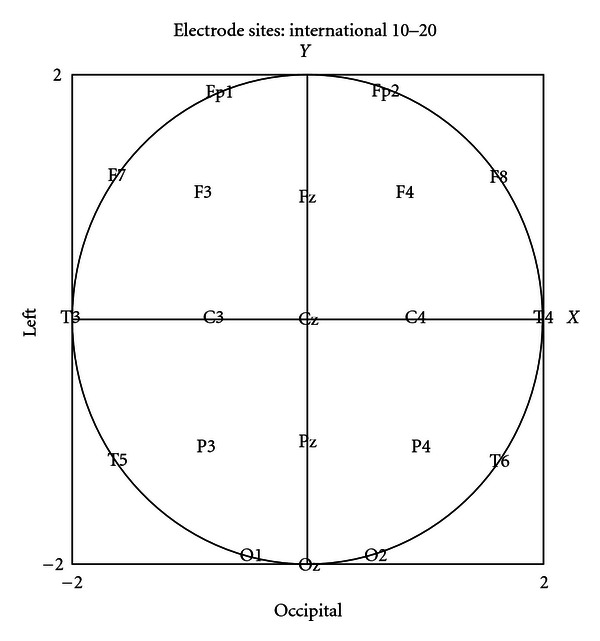
The positions of the 20 EEG channels represented as coordinates on a 2 unit by 2 unit Cartesian plane.

**Figure 2:**
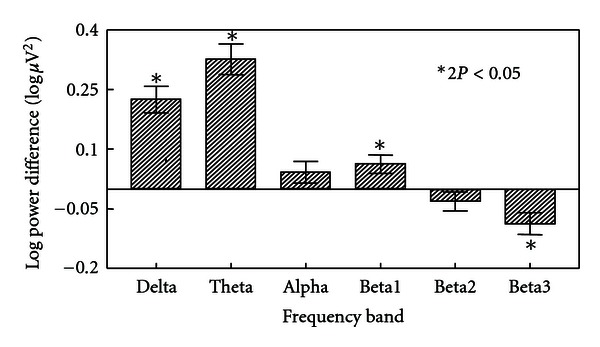
Total cranial power differences post-pre clozapine in clinical frequency bands.

**Figure 3:**
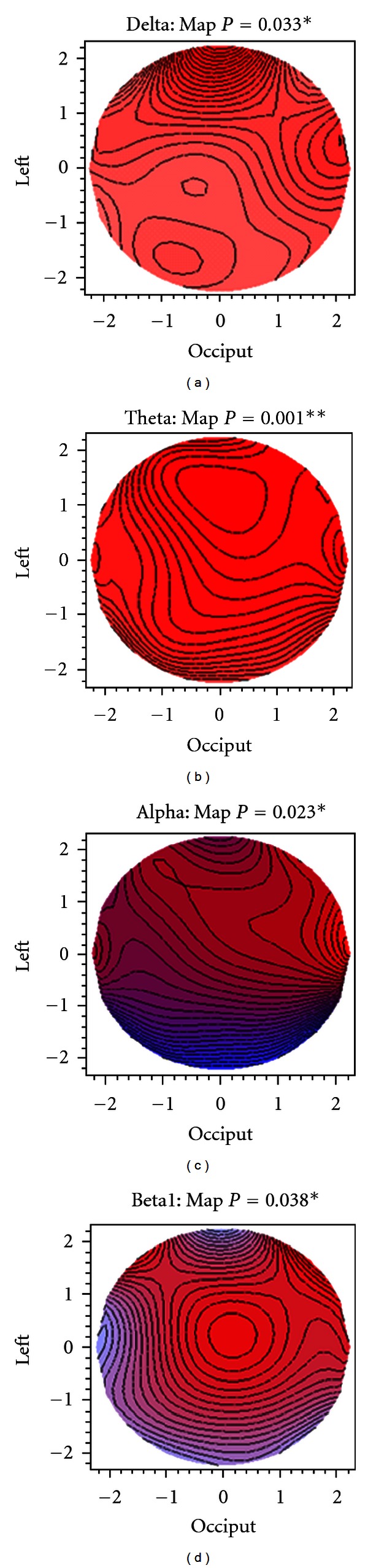
Topographic difference maps post-pre clozapine (blue: decrease, red: increase, *P* values from bootstrap *T* ^2^ tests).

**Figure 4:**
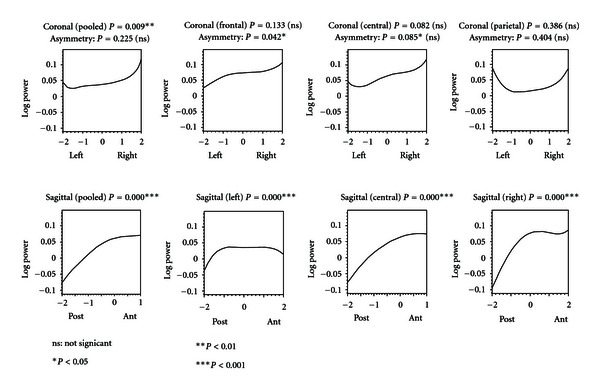
Alpha band: coronal and Sagittal sections for post-pre clozapine differences (*P* values from bootstrap *T* ^2^ tests) with cartesian plane coordinates as described in Figure 1.

The coronal sections are shown from a posterior view so that the left and right sides of the figures correspond to the left and right sides of the cranium, respectively. The sagittal sections are shown from a right-sided view so that the left and right sides of the figures correspond to the posterior and anterior regions of the cranium, respectively. The pooled and central sagittal sections are truncated at Fz (at 1.0 on the longitudinal axis shown in Figure 1) as we have no Fpz electrode and we wanted to avoid unwarranted extrapolations. The frontal coronal section passes through F7, F3, FZ, F4, F8; central: T3, C3, CZ, C4, T4; parietal: T5, P3, PZ, P4, T6. The left sagittal section passes through O1, P3, C3, F3, Fp1; the central: OZ, PZ, CZ, FZ; the right: OZ, P4, C4, F4, Fp2. The *P* values refer to the null hypothesis that log power change is evenly distributed along the section: that is to say that the difference graphs are statistically speaking, straight lines parallel to the horizontal axis. In the case of the coronal sections, there is also a test of the null hypothesis that any change in log power is symmetric about the central line: in other words that each side of the graph is a mirror image of the other.

The pooled coronal section is somewhat U-shaped, indicating greater alpha power increase in the lateral, than in the medial, areas. There is mild asymmetry in the frontal coronal section indicating greater power increase on the right side. The sagittal sections all show marked gain in frontal power at the expense of posterior power that is more pronounced for the central and right sections. Similar analyses of sagittal sections in the delta and theta frequency bands (not shown) indicate a positive laterally symmetrical gradient of power increase from posterior to anterior regions.

Thus, our major QEEG findings are that stabilized maintenance clozapine treatment causes globally increased total delta and theta power and this effect is greatest frontally. In contrast, total alpha power is unchanged but the topography shows a shift right centrofrontally.

## 4. Discussion

To our knowledge, this study reports the largest sample followed for the greatest length of time published to date employing a within subjects pre/post design before and after clozapine. Furthermore, the lengthy treatment period puts to rest any concerns that the data reflect transient effects such as the well-known sedation commonly seen when clozapine is initiated. At the time of the second data collection (mean interval 1.4 years), subjects were receiving established maintenance doses of clozapine and had adapted fully. This is of particular interest in view of the consistent reports of increased delta and theta described in the existing literature. The discovery of a right frontal shift in alpha activity is a novel finding. However, because this study was naturalistic, there was no control over concomitant psychotropic medications, and, while these were generally comparable, differences in the pattern of agents being given at the time of the second QEEG might have had some influence on our results.

There are some indications that the schizophrenic brain relies preferentially on frontal and right-sided regions for active cognitive processing. In a study of untreated schizophrenic patients, Bolsche et al. [18] showed that P300 topography resembled normals when stimuli were presented to the left ear only and that the topography shifted to the right and frontally under binaural and right only stimulation conditions. Because of the predominantly crossed nature of the auditory pathways, these data were interpreted as reflecting more compromised left hemispheric systems and in the light of the current discussion suggested that right hemisphere structures were compensating. Further support of this idea is found in the report of Weiss et al. [19]. They used functional magnetic resonance imaging in a comparison of healthy volunteers and 16 male schizophrenic patients preselected for relatively high levels of global functioning who were on established maintenance therapy of second generation antipsychotics including risperidone, olanzapine, and clozapine. During the modified Stroop task, the control group primarily activated left prefrontal cortex. Schizophrenic patients, performing equally well on the task, recruited these regions bilaterally. The authors interpreted their results as indicating that the schizophrenic subjects showed increased task-related activity in the right hemisphere.

On the other hand, right frontal structures may be involved in the generation of treatment resistant auditory hallucinations. In a PET correlation study of hallucinating and nonhallucinating patients, Copolov et al. [20] report a network of cortical activations including right medial frontal and right prefrontal regions that were associated with active hallucinations and that right thalamic activity was found in the majority of hallucinating subjects.

Scalp recorded QEEG spectral power is influenced by the activity of deeper structures such as the septal area for theta and the thalamus for alpha [21, 22]. Moreover, Danos et al. [23] found differences in alpha-related thalamo-cortical circuits between schizophrenic patients and normal controls, an observation that is of particular interest in view of the association between alpha activity and cognitive behavioural states. Thus, clozapine mediated influences on septal and thalamic areas via neuroprotective substances such as recently discovered clozapine induced Nexin gene upregulation in striatum [24] or selective reversal of stress-induced blockade of prefrontal plasticity [25] may be important clues in understanding the mechanisms and loci of its special therapeutic advantages.

## Figures and Tables

**Table 1:** Study Subjects: Clinical Characteristics (M/F=39/25).

	Mean (SD)	Minimum	Maximum
Age starting clozapine	37.3 (8.9)	22	57
Age at onset illness	20.8 (4.8)	13	32
Hospitalizations before clozapine	7.9 (9.0)	0	63
Cumulative hospital days before clozapine	505.2 (712.9)	0	3789
Preclozapine neuroleptic daily dose in CPZ equivalents (MG)	680.9 (568.6)	40	2740
Maintenance clozapine daily dose (MG)	349.5 (160.9)	50	800
Pre-post interval (years)	1.4	0.2	4.8

**Table 2:** Study subjects (M/F = 39/25): eyes closed: post-pre: *P* values.

	DF	DELTA	THETA	ALPHA	BETA1	BETA2	BETA3
Congruence	19	.033*	.001***	.023*	.038*	.085	.084
Lat × Ant.	11	.018*	<.001***	.022*	.037*	.081	.067
Coronal sections							
Pooled	6	.058	.017*	.009**	.014*	.054	.105
Asymmetry	3	.062	.088	.225	.103	.138	.262
Frontal	4	.345	.005**	.133	.025*	.050*	.027*
Asymmetry	2	.138	.121	.042*	.012*	.012*	.029*
Central	4	<.001***	.184	.082	<.001***	.001***	.003**
Asymmetry	2	.145	.154	.085	.060	.102	.223
Parietal	4	.066	.284	.386	.023*	.068	.012*
Asymmetry	2	.099	.380	.404	.491	.111	.132
Sagittal sections							
Pooled	6	.050*	<.001***	<.001***	.001**	.009**	.002**
Right	4	.304	<.001***	<.001***	<.001***	<.001***	<.001***
Centre	4	<.001***	<.001***	<.001***	<.001***	<.001***	<.001***
Left	4	.108	<.001***	<.001***	.022*	.013*	.030*
Total power	1	<.001***	<.001***	.114	.012*	.214	.003**

*P < .05,  **P < .01,  ***P < .001.

## Appendix

Within each frequency band, a total of 15 multivariate tests were applied (Table 2).

There is, however, a hierarchical structure to the sequence of tests that limits the scope for Type 1 error. The 19 degrees of freedom (df) “congruence” test asks if the overall shapes of the pre- and post-CLZ topographic maps are the same. If this test fails (i.e., *P* > .05), we would conclude that we are unable to reject the null hypothesis of congruent (i.e., same shape) pre- and post-CLZ maps. This constrains us to ask only one further question: that is, is the overall elevation of the map greater in one condition than in the other? (“Total Power” test with 1 df). All questions concerning different shapes of the sections are preempted by the failure of the “Congruence” test.

If, however, the “Congruence” null hypothesis were rejected, we may go on to ask what is the nature of the differences in shape between the two conditions. First, we ask if the differences in coronal sections are of the same degree and form along the length of the longitudinal axis (or what amount to the same thing) whether the differences in the sagittal sections are the same along the length of the transverse axis). This is tested by means of the 11 df “Laterality × Anteriority” interaction. If this fails, we must conclude that whatever differences there are before versus after in the coronal sections, they are the same wherever on the longitudinal axis they are drawn (or equivalently, that the sagittal sections are the same wherever on the transverse axis they are drawn) and we are then only permitted to test the pooled (or averaged) coronal and sagittal sections respectively. If, however, this “Lat × Ant” interaction proves significant, we ignore the pooled sections and proceed to test the three specific coronal and sagittal sections. These generally have 4 df, but, in the case of the coronal sections these can be partitioned into a 2 df test of asymmetry The former is of particular neurological interest because of the approximate lateral symmetry of structure of the brain. We may only proceed to test the 2 df coronal asymmetry differences if the 4 df coronal section difference is significant.

Applying this structural decision tree to the present data we find (Table 2) that we can reject the null hypothesis of congruent overall map shape in frequency bands delta through beta1 using a probability of a type I error (alpha) of 0.05. Thus, the 1 df “Total Power” test statistic becomes ambiguous because the distribution of power varies systematically over the cranial surface. Indeed, in the alpha band, there is both significant anterior increase and posterior decrease for a net effect on “Total Power” indistinguishable from zero (Figure 3). In contrast, we are unable to reject the null hypothesis of congruence in beta2 and beta3. Proceeding to the “Total Power” test, we find there is no reliable net change in beta2 but a reliable diminution of power in beta 3 (note that all of the 1 df “Total Power” tests are 2-tailed).

Proceeding to the “Lat × Ant” test for delta through beta1, we find we can reject the null hypothesis in all of these frequency bands, leading us to ignore the pooled coronal and sagittal sections and proceed to the individual sections which this significant “Lat × Ant” test tells us are different from each other.
